# Intralesional Steroid Injections for Management of Granulomatous Mastitis: A Systematic Review of Treatment Protocols and Clinical Outcomes

**DOI:** 10.1155/tbj/2592366

**Published:** 2025-01-21

**Authors:** J. Vercoe, N. Sedaghat, M. E. Brennan

**Affiliations:** ^1^School of Medicine Sydney, National School of Medicine, The University of Notre Dame Australia, Darlinghurst, New South Wales, Australia; ^2^Department of Surgery, Macquarie University Hospital, North Ryde, New South Wales, Australia; ^3^Westmead Clinical School, Faculty of Medicine and Health, The University of Sydney, Westmead, New South Wales, Australia

## Abstract

**Introduction:** Although idiopathic granulomatous mastitis (GM) of the breast is a benign condition, it can be locally aggressive and frequently chronic, causing significant pain and distress to the patient. Treatment often involves multiple disciplines including general practice, breast surgery/physicians, rheumatology and/or immunology. Traditional options for treatment include observation, oral steroids, methotrexate and/or surgery, all with variable outcomes. A more recent alternative treatment option involves intralesional steroid injections.

**Methods:** Using PRISMA methodology, a systematic review of intralesional steroid injection for the management of GM was conducted. Medline, PubMed, Embase and Cochrane databases were searched for original studies reporting treatment protocols and clinical outcomes, published up to the end of September 2023.

**Results:** Nine eligible studies reported outcomes in 474 patients undergoing treatment of GM with intralesional injections. All studies reported success (improvement in clinical and/or imaging appearance) with intralesional injections. Studies that had a comparison group showed statistically significantly fewer side effects compared to oral steroids or surgical management. The recurrence rate was less for intralesional injections than for other treatments in all studies except one. No studies included patient-reported outcomes.

**Conclusion:** There is consistent evidence for the safety, efficacy and low recurrence rate with intralesional steroid injections for GM. The existing literature is heterogenous with respect to injection protocols, and the optimal protocol is unclear. Future research should compare the various steroid agents and dose/frequency of administration. Future studies should include cost analysis and patient-reported outcomes to ensure that the treatment is cost-effective and acceptable to people with idiopathic GM.

## 1. Introduction

Idiopathic granulomatous mastitis (GM) is a benign, chronic inflammatory disease of the breast, first described in 1972 [1]. It is rare with an estimated prevalence of 2.4 per 100,000 women aged between 20 and 40 years [2]. Definitive diagnosis is made on histopathology from core needle biopsy [3].

The condition is characterised by noncaseating granulomas, which present as one or more erythematous, tender lumps often resulting in ulceration, abscess and sinus formation. The aetiology is unknown although the condition most commonly presents in women of childbearing age within 5 years of pregnancy, suggesting a hormonal component [4]. It is most often seen in women of Hispanic, Asian, Middle Eastern or African origin [2, 5, 6]. There is also an association with breast infection with *Corynebacterium kroppenstedtii* [7]. The entity ‘cystic neutrophilic granulomatous mastitis' has been described and appears to represent a separate entity associated with Corynebacterium [7, 8].

Despite its benign nature, GM is often locally aggressive causing significant physical pain, mental and emotional distress and reduced quality of life. GM can clinically and radiographically mimic breast cancer adding to anxiety, uncertainty and often leading to a delay in diagnosis [9]. Due to its relatively rare incidence, there is often a delay in patients being referred to treatment centres with experience in managing the condition.

Presently, there is no ‘gold-standard' approach to the management of GM. The traditional options are conservative/supportive management (pain relief, dressings and aspirating abscesses), oral immunosuppressive therapy (prednisone and/or methotrexate) and surgical excision. A consensus statement published in 2022 discussed these options for management and recommended an individualised approach based on the clinical features at the time of presentation [10].

The clinical course is unpredictable, and inflammatory lesions tend to relapse in the same or different areas of the breast during treatment and healing. The success rate following the various treatment options is highly variable, and adverse systemic effects are particularly common in patients receiving oral prednisone and/or methotrexate. When managed conservatively, the lesions of GM may take 12 months or more to heal during which time more lesions may arise in a chronic remitting/relapsing pattern. Post-treatment recurrence of GM treated with oral medication approaches 50% [11].

Surgical excision is not always feasible as the condition may be diffuse, affecting two or more quadrants of the breast. When excised, there is an estimated recurrence rate of 13% [12]. Poor cosmetic outcomes following surgery have led to a focus on medical management.

A more recent treatment approach utilises ultrasound-guided intralesional steroid injections. Early results suggest complete resolution of the lesion/s with sustained regression rates and negligible adverse effects [13–15]. Local injection of steroids allows for targeted dosing to the affected tissues, thus reducing systemic adverse effects of oral immunosuppression. This option has been included in a consensus statement [10].

The aims of this systematic review were (1) to examine the literature reporting intralesional steroid injections for GM to describe the protocols, efficacy and adverse effects of the treatment and (2) to use this information to develop evidence-based recommendations for clinicians considering steroid injections for their patients.

## 2. Materials and Methods

This review was conducted using the Preferred Reporting Items for Systematic Reviews and Meta-Analyses (PRISMA) methodology [16].

### 2.1. Eligibility Criteria

The inclusion criteria were English-language full-text studies in peer-reviewed journals, reporting original data on an intralesional steroid protocol and/or outcomes of intralesional steroid treatment in GM. All study designs were eligible (observational or randomised, prospective or retrospective). A comparison group was not essential.

The exclusion criteria included papers not evaluating intralesional steroid injections as a stand-alone method of treatment. Duplicated studies, single case reports, review articles, letters and conference abstracts were ineligible.

### 2.2. Information Sources and Search Strategy

Medline, PubMed, Embase and Cochrane databases were searched from inception to the end of September 2023, filtered to include the English language only. The databases were searched using the following terms: ‘granulomatous mastitis' OR ‘idiopathic granulomatous mastitis' OR ‘granulomatous lobular mastitis' OR ‘cystic neutrophilic granulomatous mastitis' AND ‘injection' OR ‘intralesional' OR ‘intralesional injection' AND ‘corticosteroid' OR ‘triamcinolone'. Reference lists of eligible papers were also searched to identify additional relevant studies.

### 2.3. Selection Process

Studies identified in the initial search were imported into EndNote, and duplicates were removed. Titles and abstracts were screened for eligibility by two reviewers (JV/MB) independently. Full-text papers of eligible abstracts were screened again by both reviewers.

### 2.4. Data Collection, Data Items and Data Synthesis

Data extraction was performed by one author (JV) and checked for accuracy by the other (MB). Disagreement was resolved by discussion and consensus. Data were stored in an Excel spreadsheet.

The following data items were collected: author, publication date and journal, country of origin, study design, aim, methodology, patient population, comparison group, confirmation of GM diagnosis, injection schedule, drug and dose, measurement of response, results and conclusions.

Information about the patient population included eligibility for the study and the number and characteristics of recruited participants for each study. Outcome data analysed the response to treatment using individual study definitions for ‘complete', ‘partial' and ‘no' response. Other data included lesion size (clinical or ultrasound), clinical appearance, patient-reported symptoms and side effects, duration to complete recovery and recurrence rate where included.

Each study was examined, and the methodology and results of all studies were compared. Data were reported in a narrative form. Due to the nature of the data (few studies, small studies with qualitative data) and the heterogeneity of the studies, statistical analysis and meta-analysis were not considered appropriate.

### 2.5. Risk-of-Bias Assessment

Risk of bias was assessed using the Joanna Briggs Institute's risk-of-bias critical appraisal tool for cohort studies [17]. Each study was assessed against 11 criteria (for cohort studies) or 13 criteria (for randomised trials). A rating of yes/no/unclear/not applicable was given for each item. Assessment was performed by one author (JV) and checked by the other (MB), and discordance was resolved by discussion and consensus. Studies were considered to be ‘low risk of bias' if they received no more than one ‘no' or ‘unclear' answer for criteria. They were ‘moderate risk' if there were two or three ‘no' or ‘unclear' answers and ‘high risk of bias' if there were more than three.

## 3. Results

### 3.1. Study Selection

**Figure 1:**
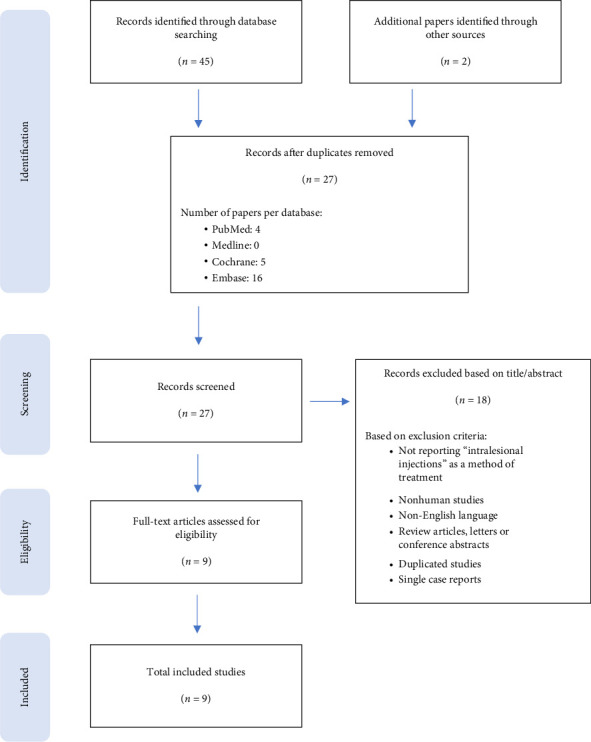
Flow diagram of the review process (PRISMA). PRISMA, Preferred Reporting Items for Systematic Reviews and Meta-Analyses.

The PRISMA flowchart is shown in Figure 1. The initial search identified 45 papers that were imported to EndNote. After removing duplicates, 27 articles were screened by title and abstract. When the inclusion and exclusion criteria were applied to full-text papers, nine eligible studies were identified for inclusion in data synthesis [13–15, 18–23]. These reported outcomes in 474 patients undergoing treatment of GM with intralesional injections.

### 3.2. Study Characteristics

Characteristics of the eligible studies are shown in Table 1. There were six studies from Turkey [13, 14, 18, 21–23] and one each from Iran [19], South Korea [20] and USA [15].

Three studies utilised methylprednisolone acetate for intralesional injections [13, 18, 22], five evaluated the effect of triamcinolone acetonide [14, 15, 20, 21, 23], and one utilised betamethasone acetate [19].

Of the six studies that examined intralesional steroid injections against a comparator, three compared to oral steroid therapy alone [18, 21, 23], one compared to surgical resection [14], one to surgical resection and observation [15] and one compared to oral therapy, and local injection and oral therapy combination [19].

There were four prospective [13, 18, 19, 23] and five retrospective studies [14, 15, 20–22]. Two prospective studies were randomised: one via sealed envelope randomisation [23] and the other single-blind [19].

All studies utilised histopathology to confirm the diagnosis of GM in study participants.

Variables measuring outcome were similar across all nine studies: all included data on response to treatment and side effects. Six analysed recurrence rates [14, 18–22] and four discussed time to remission [15, 18–20].

### 3.3. Risk of Bias

Using JBI tools [17], there were seven studies assessed as ‘low risk of bias', and all were cohort studies [13–15, 18, 20–22], and two assessed as ‘high risk of bias', both randomised trials [19, 23].

### 3.4. Treatment Efficacy

Table 2 shows the treatment results of all eligible studies. Of the four studies that compared intralesional injections to oral steroid therapy, all reported a higher success rate with injections [18, 19, 21, 23].

Success was demonstrated by a ‘complete' response, which was defined by the complete clinical and radiological absence of disease [18, 19, 21, 23]. Karami et al. defined excellent and good control as those with a resolution of > 90% of signs and symptoms with and without recurrence, respectively [19].

The reported success rates of intralesional steroid injection from other studies included 95.3% compared to 87% for oral therapy [18], 93.5% complete response compared to 71.9% for oral therapy [21] and 88.2% clinical response compared to 76.4% [23]. One study reported the time to complete remission of 3.17 months for intralesional steroid treatment compared to 6.37 months for systemic oral therapy [19]. The same study by Karami et al. compared intralesional injections to combination injection and oral steroid therapy; it was observed that the time to remission for combined therapy was 4.33 months. Approximately 90% of patients achieved excellent or good control after four courses of intralesional steroid injection [19].

Two studies compared intralesional steroid injection to surgical intervention [14, 15]. One found a higher rate of complete response without recurrence within the follow-up period with intralesional steroid injection (94.5% vs. 75.8%) [14], while the other reported no difference as all patients, regardless of the treatment arm, achieved clinical resolution [15]. There was a statistically significant reduction in pain post-treatment for those receiving injections compared to surgery [14], while the median time to complete response was 2 months with steroid injections and 0.5 months with surgery [15].

Of the remaining three studies, without comparators, two had a complete response rate of 100% within two treatment courses—83.3% with one course of injections and one patient requiring a second course [22] and 89.3% and 10.7% complete and partial responses, respectively [13]. In the first, complete response was defined as remission of clinical and radiological features of GM [22] while the second did not define complete and partial response [13]. The final study reported a mean time to complete remission of 115.3 days, and the lesion size decreased significantly after one injection (*p* < 0.001) [20].

### 3.5. Recurrence

Recurrence rates were discussed in six of the studies [14, 18–22]. One study reported no recurrence after intralesional steroid injection [14]. The other five studies identified recurrence after intralesional therapy: 4.7% vs 12.5% for systemic oral steroids [18], 8.7% vs 46.9% for systemic oral steroids [21], 16.4% vs 3.3% for systemic oral steroids [19] and one patient in a cohort of six required follow-up treatment [22].

This study published their follow-up management and reported that a third dose of intralesional methylprednisolone acetate was successful in achieving complete remission [22]. Another study reported that six patients had recurrence within 8 months of intralesional injection, but all were managed with reinjection, and no recurrence was reported within the follow-up period [20].

There was significant variability in the mean follow-up duration between studies. Six of the nine studies reported on the duration of follow-up: 17.5 months [21], 19.5 months [22], 6 months [23], 11.8 months [13], 10 months [19] and 16.6 months [20].

### 3.6. Adverse Effects

There were no adverse effects identified from six of the nine studies reporting adverse effects of intralesional steroid injections [13, 14, 19, 20, 22, 23].

Of the studies that identified adverse effects, these were consistent with steroid-related symptoms and changes including joint pain, nausea, weight gain, hair loss and skin atrophy/thinning of the skin [15, 18, 21]. The rate of adverse effects, however, were significantly lower in patients receiving intralesional steroid injections compared to oral systemic therapy (14.3% vs. 81.3%, *p* < 0.001) [18]. The adverse effects observed on oral systemic therapy included oedema, weight gain, hirsutism, restlessness, acne, menstrual irregularity, palpitations, euphoria and glaucoma [15, 18, 21].

### 3.7. Injection Protocol

The injection protocol varied significantly between the studies (Table 3). The studies evaluated different steroid preparations (triamcinolone, methylprednisolone and betamethasone), different doses and steroid mixed with or without lidocaine. The frequency of injection varied from weekly to four weekly, and the duration of treatment varied from a single injection to 11 injections. Most continued treatment until resolution. The response rate to all protocols was very high, and there was no clear trend to suggest that one was more efficacious than the others.

## 4. Discussion

This systematic review identified nine studies that evaluated intralesional steroid injection to treat idiopathic GM. Despite being a benign condition, GM is locally aggressive and causes significant long-term pain and distress for affected patients. The traditional treatments of oral steroids, methotrexate and/or surgery may be successful [10, 24, 25]; however, they are also associated with significant adverse effects and do not always result in the resolution of the symptoms. The option to treat with intralesional steroid injection is appealing if it is efficacious with lower morbidity. When compared to observation, oral steroids and surgical excision, there was a consistent observation across studies that intralesional steroid injections are effective with low morbidity.

A number of different injection regimens were tested across the nine papers comprising three different steroid types injected, varied steroid dosages and injection schedules. Given the high ‘success' rate (defined in different ways) of steroid injections, it is unclear which regimen is ‘best'. Four-weekly injections until complete response or recovery were employed in the majority of papers, but whether this constitutes optimal scheduling cannot be determined. One of the nine papers used betamethasone acetate injections and was the only study to record higher recurrence rates with intralesional steroids compared to other treatments (16.4% vs. 3.3% for oral steroids) [19]. Further research is needed to qualify a possible link between this outcome and the use of betamethasone acetate as well as the distinction in dose (3 mg/mL compared to 40 mg/mL employed in the remaining seven papers) [19]. However, it may be that no single agent is superior to others.

The adverse effects of intralesional injections were observed to be uncommon and mild. Most women treated in these studies did not experience any adverse effects. Some of the reported side effects, such as nausea and joint pain, are not typically associated with steroid injections when used for musculoskeletal conditions so they require further evaluation.

Two of the studies were prospective randomised control trials [19, 23]. While this study design is more robust, these were assessed as ‘high risk of bias' compared to the cohort studies that were all ‘low risk'. The results were similar across all nine studies in this review so risk-of-bias classification is not associated with a higher likelihood of successful treatment with any particular regimen.

Time to complete recovery ranged from 1 month to 5 months with recurrence reported in six studies [14, 18–22]. Two studies reported the use of repeat intralesional steroid treatment for recurrence [20, 22]. It is not clear what constitutes best practice when managing recurrences and the safety of repeating intralesional injections is unclear. Despite the efficacy of treatments, it is evident that idiopathic GM remains a chronic condition. Although intralesional injections appear to offer advantages over oral therapies and surgery, they do not result in immediate resolution and a protracted clinical course remains common.

None of the studies in this review specifically addressed the entity of cystic neutrophilic GM. It is therefore unknown whether this condition responds differently to steroid injections and whether injections should be used in conjunction with antibiotic therapy or after the infection is treated. It is likely that cases of the cystic neutrophilic subgroup of GM were included in the cohorts, but the outcomes were not reported separately. This may be due to the cases not being recognised or the authors may not have considered separate reporting warranted. The efficacy of intralesional injections is likely to have been demonstrated; however, the magnitude cannot be determined from these data.

While this analysis posits the clinical efficacy of intralesional steroid injections, there is a lack of data regarding patient opinion and preferences with respect to treatment. While there is an assumption that patients would prefer injections over oral therapy or surgery due to the improved morbidity profile, this has not been evaluated. Some may prefer oral medication for a longer duration rather than repeated clinic presentations. It is also possible that some patients are averse to receiving injections due to fear of needles or other factors. A comparison of the costs of the various treatments has also not been explored, and this has the potential to heavily influence both patient and provider appeal.

This review has strengths and limitations. The strengths include the robust PRISMA methodology with broad search criteria across multiple databases, making it unlikely that eligible studies were missed. The main limitation is the heterogeneity of the nine studies. While they all employ intralesional steroid injections, they use different steroid preparations, number of injections and dose intervals. They measure efficacy differently, and the randomised studies were of poor quality (high risk of bias).

## 5. Conclusions

The nine studies included in this review all reported the success of intralesional steroid injections for the treatment of idiopathic mastitis. Despite the limited number of studies and their heterogenous protocols, there is consistent evidence for safety, efficacy and low recurrence with injections. The morbidity of oral steroid treatments and surgery further suggests that intralesional steroid injections can be recommended as a valuable treatment option to consider as an alternative to these proven treatments of surgery and/or systemic therapies.

There remains insufficient evidence to advocate for one injection protocol over another. There were no trials that directly compared different steroid injection agents; however, the highest recurrence rate was reported in the study employing betamethasone acetate. Future research should look to compare the different injection protocols—specifically steroid agent, dose and frequency, and analyse the costs associated with the various treatments. Patient-reported outcomes with respect to acceptability of injections, impacts on quality of life and pain are highly relevant, and this should also be a space for further investigation.

## Figures and Tables

**Table 1:** Characteristics of studies reporting outcomes of the use of intralesional steroid injections for the management of GM.

First author, year	Title	Journal	Country, year	Study design	Population (*n*)	Intervention	Comparator	Outcomes measured
Alper, 2022	Comparison of the efficacy of systemic versus local steroid treatment in idiopathic granulomatous mastitis: A cohort study	Journal of Surgical Research	Turkey, 2015–2019	Prospective	• 58 female patients • Presented to hospital outpatient clinic • Diagnosed with GM via histopathology	Intralesional steroid injection *methylprednisolone acetate* 40 mg/mL (*n* = 42)	Oral steroids (*n* = 16)	• Response to treatment • Time to remission • Recurrence rates • Side effects

Erturk, 2021	Local steroid treatment: An effective procedure for idiopathic granulomatous mastitis, including complicated cases	Journal of Investigative Surgery	Turkey, 2017–2019	Retrospective	• 86 female patients • Over 18 years of age • Presented to Kocaeli University Research and Application Hospital • Diagnosed with GM via histopathology	Intralesional steroid injection *triamcinolone acetonide* 40 mg/mL (*n* = 38)	Surgical resection (*n* = 48)	• Response to treatment • Recurrence rates • Side effects • Pain scores

Tang, 2020	Granulomatous mastitis: Comparison of novel treatment of steroid injection and current management	Journal of Surgical Research	USA, 2003–2017	Retrospective	• 49 female patients • Presented to a safety-net county hospital • Diagnosed with GM via histopathology	Intralesional steroid injection *triamcinolone acetonide* 40 mg/mL (*n* = 12)	Observation (*n* = 28) Surgical resection (*n* = 9)	• Response to treatment • Time to remission • Side effects

Toktas, 2021	A novel first-line treatment alternative for noncomplicated idiopathic granulomatous mastitis: Combined intralesional steroid injection with topical steroid administration	Breast Care	Turkey, 2015–2018	Retrospective	• 78 female patients • Presented to breast centres across Turkey • Diagnosed with GM via histopathology	Intralesional steroid injection *triamcinolone acetonide* 40 mg/mL with topical steroid administration (*n* = 46)	Oral steroids (*n* = 32)	• Response to treatment • Recurrence rates • Side effects • Complication rates

Toktas, 2021	Treatment results of intralesional steroid injection and topical steroid administration in pregnant women with idiopathic granulomatous mastitis	Journal of Breast Health	Turkey, 2017–2019	Retrospective	• 6 female patients • Pregnant—third trimester • Presented to a breast clinic • Diagnosed with GM via histopathology	Intralesional steroid injection *methylprednisolone acetate* 40 mg/mL (*n* = 6)	None	• Response to treatment • Recurrence rates • Side effects

Yildirim, 2021	Comparison of the efficiency of systemic therapy and intralesional steroid administration in the treatment of idiopathic granulomatous mastitis. The novel treatment for granulomatous mastitis	Annali Italiani di Chirurgia	Turkey, Unknown	Prospective RCT	• 36 female patients • Presented to the breast unit at Health Sciences University Turkey • Diagnosed with GM via histopathology	Intralesional steroid injection *triamcinolone acetonide* 40 mg/mL (*n* = 17)	Oral steroids (*n* = 19)	• Response to treatment • Side effects

Alper, 2020	The evaluation of the efficacy of local steroid administration in idiopathic granulomatous mastitis: The preliminary results	Breast Journal	Turkey, 2015–2018	Prospective	• 28 female patients • Presented to a tertiary university hospital • Diagnosed with GM via histopathology	Intralesional steroid injection *methylprednisolone acetate* 40 mg/mL (*n* = 28)	None	• Response to treatment • Side effects

Karami, 2022	The effectiveness of local steroid injection for the treatment of breast-limited idiopathic granulomatous mastitis: A randomized controlled clinical trial study	BMC Women's Health	Iran, 2020	Prospective RCT	• 118 female patients • Referred to Centre for Breast Diseases, South Iran • Diagnosed with GM via histopathology	Intralesional steroid injection *betamethasone acetate* 3 mg/mL (*n* = 31)	Oral steroids (*n* = 30). Combined oral and intralesional injection (*n* = 38)	• Response to treatment • Time to remission • Recurrence rates • Side effects

Kim, 2016	Usefulness of ultrasound-guided intralesional steroid injection in management of idiopathic granulomatous mastitis	Journal of Surgical Ultrasound	South Korea, 2012–2016	Retrospective	• 15 female patients • Diagnosed with GM via histopathology	Intralesional steroid injection *Triamcinolone acetonide* 40 mg/mL (*n* = 10) Intralesional steroid injection *Triamcinolone acetonide* 40 mg/mL with oral prednisolone (10 mg) (*n* = 5)	None	• Response to treatment (size of lesion) • Time to remission • Recurrence rates • Side effects

**Table 2:** Methods and results from studies reporting outcomes of the use of intralesional steroid injections for management of GM.

First author, year	Intervention	Methods	Duration of treatment	Duration of follow-up	Side effects	Recurrence	Other results	Conclusions
Alper, 2022	Intralesional steroid injection *methylprednisolone acetate* 40 mg/mL (*n* = 42)	• Intralesional steroid injection at 4-week intervals • Oral doses daily with first follow-up at 3 months • Response measured via imaging (US, MRI)	• Median duration of treatment was 5 months for local and 3 months for systemic treatment • Local injection therapy was repeated once to 11 times and continued until complete recovery—the median number of injections was four • The dose was doubled in patients receiving local injections without recovery after 1 year	• Not reported	• Six patients (14.3%) receiving local treatment developed steroid-related side effects (joint pain, nausea, weight gain, hair loss) • 81.3% of patients receiving systemic treatment developed side effects • Statistically significant fewer side effects than systemic treatment group	• Recurrence rate of 4.7% for local treatment and 12.5% for systemic treatment • There was no statistically significant difference (*p*=0.06)	• Local treatment success rate of 95.3%, systemic treatment success rate of 87% • A median dose of 140 mg was administered in the local treatment group • A median dose of 3810 mg was administered in the systemic treatment group	• Local treatment was as effective as systemic treatment therapy • Local treatment achieved such results with a lower steroid dose and fewer side effects • Local treatment can be considered a new therapeutic protocol

Erturk, 2021	Intralesional steroid injection *triamcinolone acetonide* 40 mg/mL (*n* = 38)	• Intralesional steroid injection at 4-week intervals with topical triamcinolone acetonide administered once a day for 1 month • Surgical treatment consisted of local excision, wide excision, mastectomy • Response measured via imaging (US) • Treatment continued until complete or partial recovery	• This procedure was repeated monthly until the treatment result was accepted as complete recovery or partial recovery • Partial recovery was recorded if the lesion size remained the same after two consecutive sessions • In order to achieve complete recovery, the median number of sessions was 2 for 45 lesions < 3 cm in size and 3 for 25 lesions of > 3 cm in size (*p*=0.002)	• Not reported	• No local or systemic steroid-related side effects were observed	• No recurrence in any patients receiving local steroid injection • Recurrence in 15 (31.2%) patients who received surgical treatment—this was significantly higher (*p* < 0.001)	• In the 38 patients who received local treatment, 70 of 72 (97.2%) lesions had complete response • Two lesions were classified as partial response to treatment (complaint persisted or partial response) • Complete recovery was achieved in 36 of 38 patients (94.5%) • In the local treatment group, 14 patients described their pain as mild and 24 described no pain • In the surgical group, 1 patient described their pain as severe and 24 described their pain as moderate • Statistically significant reduction in pain post-treatment in steroid injection group vs surgery group • The median treatment cost of local injection and surgical treatment was 21 USD and 198.50 USD, respectively	• Local steroid injections are associated with full resolution • Local steroid injections are less expensive, better reduce pain and have lower recurrence rates as compared to surgery • Local steroid injections should be first-line treatment

Tang, 2020	Intralesional steroid injection *triamcinolone acetonide* 40 mg/mL (*n* = 12)	• Intralesional steroid injection, 40 mg/mL • Surgical treatment involved excision of gross disease with small normal tissue margin • Response measured via resolution of symptoms	• Treatment continued until resolution of symptoms was achieved • Time to resolution was 3–18 months for observation, 0.5–3.3 months for steroid injection and 0.5–2 months for surgical excision	• Not reported	• One patient receiving intralesional steroid injections suffered localised skin atrophy	• Not reported	• Median time to resolution was 11.5 months, 2 months and 0.5 months for observation, steroid injection and surgical excision, respectively (*p* < 0.001) • Both steroid injection and surgical excision resolution time was significantly decreased from the observation cohort (*p*=0.002, *p*=0.001, respectively) • There was no difference in resolution time between steroid injection and surgical cohorts (*p* > 0.999) • All patients who underwent steroid injection and surgical incision had resolution	• Intralesional steroid injections are effective with minimal side effects • More studies are needed to determine which treatments are best for which groups of people

Toktas, 2021	Intralesional steroid injection *triamcinolone acetonide* 40 mg/mL with topical steroid administration (*n* = 46)	• Intralesional steroid injection at 4-week intervals • Topical steroid administration on the skin of the affected region of the breast twice a day on every other day for 1 month • Oral treatment administered once a day for 1 month • Response measured via imaging and resolution of symptoms	• Patients with ‘complete response' were enlisted to follow up after 1 month • Patients with ‘partial response', ‘no response' and ‘worsening disease' received a second and, if necessary, third month of treatment • Monthly check-ups were performed until resolution	• The mean follow-up time for patients receiving local injections was 17.5 months compared to 23.2 months for those receiving oral steroids	• Complication rates were similar between groups (*p*=0.16) • Thinning of the skin was observed in one patient receiving local injection and topical therapy • Three patients receiving oral steroids had systemic side effects (hirsutism and weight gain)	• Recurrence rates were significantly lower in those receiving steroid injections—8.7% compared to 46.9% in the oral steroid cohort (*p*=0.001)	• 43 patients (93.5%) receiving intralesional steroid injections achieved partial or complete response compared to 23 patients (71.9%) receiving oral systemic steroid therapy after 3 months • Treatment response was significantly higher in patients receiving local injections compared to oral steroids (*p*=0.012) • The need for surgical treatment was significantly less for those receiving steroid injections—2.2% vs. 9.4% (*p*=0.001)	• Combined steroid injection with topical steroids is as effective at treating IGM as systemic steroid treatment with less complications and side effects • Recommendation for this combination therapy to be used as first-line therapy in patients with noncomplicated IGM

Toktas, 2021	Intralesional steroid injection *methylprednisolone acetate* 40 mg/mL (*n* = 6)	• Intralesional steroid injection at 4-week intervals with topical administration of 0.125% prednisolone twice a day, on alternate days for 4 weeks • Response measured via imaging and resolution of symptoms	• Treatment response was evaluated clinically and radiologically after 2 weeks and again after 1 month • Complete response was achieved by all patients at the end of the second course of treatment	• The mean follow-up time was 19.5 months	• Neither topical nor systemic side effects of corticosteroids were observed in any patient	• During follow-up, one patient experienced a recurrence at 4 months postbirth—she received a third steroid injection and achieved complete response	• Five patients (83.3%) achieved complete response after one steroid injection • One patient achieved complete response after a second steroid injection	• Intralesional steroid injections required a shorter duration of treatment • They had fewer side effects and less need for surgery compared to systemic steroid therapy • Recurrence rates were lower with intralesional steroid injections

Yildirim, 2021	Intralesional steroid injection *triamcinolone acetonide* 40 mg/mL (*n* = 17)	• Intralesional steroid injection at 1-week intervals • Oral treatment once per day for 1 month • Lesions < 5 cm received 20 mg/mL, lesions > 5 cm received 40 mg/mL • Response measured via imaging and physical examination at 1, 3 and 6 months	• Of the local injection group, 13 patients received single and four patients received multiple injections (these patients had three or more lesions)	• The follow-up period ended 6 months after the treatment was completed	• Two of the patients receiving systemic therapy complained of side effects (weight gain)	• Not reported	• 10 patients receiving local treatment had clinical response within 1 month, 15 had clinical response within 3 months and 15 within 6 months (88.2%) • 10 patients receiving systemic treatment had clinical response within 1 month, 13 within 6 months • Two of the 17 patients receiving local treatment were considered unresponsive to treatment after 6 months, both had multifocal lesions • Four of the patients receiving systemic oral treatment did not respond to treatment by 6 months	• The treatment response was higher in the local injection group as compared to those receiving oral steroids • The result was not statistically significant—clinically or radiologically—between the two groups

Alper, 2020	Intralesional steroid injection *methylprednisolone acetate* 40 mg/mL (*n* = 28)	• Intralesional steroid injection with follow-up intervals of 3–4 weeks • Response measured via imaging and resolution of symptoms	• Patients received two, three, four, five or seven injections at intervals of 3–4 weeks	• The average follow-up period was 11.8 (5–20) months	• No steroid side effects or complications due to local application were observed	• Not reported	• Partial response was obtained in three patients (10.7%), and complete response was obtained in 25 patients (89.3%) • In patients who received two injections (*n* = 8), two achieved partial response and six achieved full recovery • In patients who received three injections (*n* = 10), one achieved partial response and nine achieved full recovery • In patients who received four, five and seven injections (*n* = 10), all patients achieved complete recovery	• Local injection treatment seems to be effective, safe and complication-free • Recommendation for local steroid treatment to be included in IGM treatment protocol

Karami, 2022	Intralesional steroid injection *betamethasone acetate* 3 mg/mL (*n* = 31)	• Intralesional steroid injection weekly, one–four times • Oral therapy was administered once daily and tapered at 2 weeks • Response measured via resolution of symptoms	• The time to complete remission was 1–6 months for local injection and combined therapy and 6–9 months for the oral systemic group	• Total follow-up period was 10 months	• Four patients in combined therapy group and three patients in systemic therapy group had systemic side effects • No side effects were observed for the local steroid injection group	• Recurrence rate was 16.4%, 13.2% and 3.3% in the injection, combined and systemic oral therapy groups, respectively • Of the 69 patients who received local injections, five developed disease progression after the fourth steroid injection (two in combined therapy group, three in the local injection group)—all withdrew and switched to oral steroid therapy	• The mean time to half regression was 1 month in the injection group and 6.33 months in the systemic oral therapy group • The time to complete regression was 3.17 months, 4.33 months and 6.37 months in the injection, combined therapy and systemic oral therapy groups, respectively • The initial response to local injection was rapid in the combined and injection group—the mass lesion shrank significantly after four injections in these groups	• Local steroid injection is as effective as systemic immunosuppressive therapy in the treatment of IGM • Local therapy has significantly fewer side effects

Kim, 2016	Intralesional steroid injection *triamcinolone acetonide* 40 mg/mL (*n* = 10) and intralesional steroid injection *triamcinolone acetonide* 40 mg/mL with oral prednisolone (10 mg) (*n* = 5)	• Intralesional steroid injections every one or 2 weeks, repeated until resolution of symptoms • Oral prednisolone daily for patients with multiple, large or painful abscesses • Response measured via imaging and resolution of symptoms	• The mean individual remission time of the lesions (*n* = 30) was 37.5 days • The mean complete remission time was 115.3 days • All IGM lesions responded to intralesional injection	• The mean follow-up time was 16.6 months	• There were no complications related to intralesional steroid injection under ultrasound guidance	• In 6 patients, the IGM lesions recurred at the same sites less than 8 months after intralesional injection—these were managed with reinjection • There were no recurrences during follow-up	• The mean size of the lesions was significantly reduced after the first and second injections (*p* < 0.001), 13.7 mm and 8.4 mm respectively compared to 22.2 mm pretreatment • The mean size of the lesions after three, four and five injections was 7.8 mm, 6.4 mm and 6.5 mm, respectively • There were no complications related to intralesional steroid injections	• Intralesional steroid injection appears to be effective in treating IGM

**Table 3:** Outcomes of treatment protocols for intralesional steroid injections for the management of idiopathic granulomatous mastitis.

First author, year	Injection drug and dose	Comparator	Duration of treatment	Recurrence
Alper, 2022	*Methylprednisolone acetate* 40 mg/mL (1 mL with 5 mL saline)	Oral steroids	• Injection repeated every 3–4 weeks on 1–11 occasions and continued until complete recovery • Median number of injections was 4	• Recurrence rate of 4.7% for local treatment and 12.5% for systemic treatment Not significant (*p*=0.06)

Toktas, 2021	*Methylprednisolone acetate* 40 mg/mL (Nil)	None	• Treatment response was evaluated clinically and radiologically after 2 weeks and again after 1 month • Complete response was achieved by all patients at the end of the second course of treatment	• During follow-up, one patient experienced a recurrence at 4 months postbirth—she received a third steroid injection and achieved complete response

Alper, 2020	*Methylprednisolone acetate* 40 mg/mL (1 mL with 5 mL saline)	None	• Injection repeated every 3–4 weeks until complete response achieved • Patients received two, three, four, five or seven injections	• Not reported

Erturk, 2021	*Triamcinolone acetonide* 40 mg/mL (1:2 to 1:4 steroid: Saline dilution dependent on number and size of lesions, 1 unit of 2% lidocaine added)	Surgical resection	• Injection repeated once per month until the treatment result was accepted as complete recovery or partial recovery • Partial recovery was recorded if the lesion size remained the same after two consecutive sessions • Median number of injections = 2 for lesions < 3 cm in size and 3 for lesions of ≥ 3 cm	• No recurrence in any patients receiving local steroid injection • Recurrence in 15 (31.2%) patients who received surgical treatment—this was significantly higher (*p* < 0.001)

Tang, 2020	*Triamcinolone acetonide* 40 mg/mL (2–4 mL with 2 units 1% lidocaine)	Observation Surgical resection	• Frequency of injection not reported • Injections continued until resolution of symptoms was achieved • Time to resolution was 3–18 months for observation, 0.5–3.3 months for steroid injection and 0.5–2 months for surgical excision	• Not reported

Toktas, 2021	*Triamcinolone acetonide* 40 mg/mL with topical steroid administration (Nil)	Oral steroids	• Injection repeated once per month until treatment was accepted as complete response • Patients with ‘complete response' were enlisted to follow up after 1 month • Patients with ‘partial response', ‘no response' and ‘worsening disease' received a second and, if necessary, third month of treatment • Monthly check-ups were performed until resolution	• Recurrence rates were significantly lower in those receiving steroid injections—8.7% compared to 46.9% in the oral steroid cohort (*p*=0.001)

Yildirim, 2021	*Triamcinolone acetonide* 40 mg/mL (Diluted with saline and adrenaline-free lidocaine—unreported ratio)	Oral steroids (*n* = 19)	• Injections were given at intervals of 1 week • Of the local injection group, 13 patients received single, and four patients received multiple injections • All patients receiving multiple injections had three or more lesions	• Not reported

Kim, 2016	*Triamcinolone acetonide* 40 mg/mL or *triamcinolone acetonide* 40 mg/mL with oral prednisolone (10 mg) (4 mL of 40 mg triamcinolone with 2% lidocaine)	None	• Injections were performed once every 1–2 weeks and repeated until resolution of symptoms and ultrasonographic findings • The mean individual remission time of the lesions (*n* = 30) was 37.5 days • The mean complete remission time was 115.3 days • All IGM lesions responded to intralesional injection	• In 6 patients, the IGM lesions recurred at the same sites less than 8 months after intralesional injection—these were managed with reinjection • There were no recurrences during follow-up

Karami, 2022	*Betamethasone acetate* 3 mg/mL (3 mg betamethasone acetate with 3 mg/mL betamethasone disodium phosphate)	Oral steroids (*n* = 30), combined oral and intralesional injection (*n* = 38)	• Injections were repeated weekly • Clinical improvement including closure of fistula orifices, disappearance of inflammatory signs and/or skin erosions, and healed skin ulceration was regarded as criteria to terminate treatment • The time to complete remission was 1–6 months for local injection and combined therapy and 6–9 months for the oral systemic group	• Recurrence rate was 16.4%, 13.2% and 3.3% in the injection, combined and systemic oral therapy groups, respectively • Of the 69 patients who received local injections, five developed disease progression after the fourth steroid injection (two in the combined therapy group and three in the local injection group)—all withdrew and switched to oral steroid therapy

## Data Availability

The systematic review data used to support the findings of this study are included within the article.
